# Light-Driven On-Surface Synthesis: Mechanisms, Strategies, and Architectures

**DOI:** 10.3390/nano16090534

**Published:** 2026-04-28

**Authors:** Yinghui Fu, Ying Han, Jiuan Gong, Jiahui Li, Yiwen Wang, Chao Yan, Rengang Wan, Xin Zhang, Jianzhi Gao

**Affiliations:** School of Physics and Information Technology, Shaanxi Normal University, Xi’an 710119, China

**Keywords:** molecular self-assembly, on-surface synthesis, photochemistry, scanning tunneling microscopy

## Abstract

Molecular on-surface photochemistry has emerged as a promising alternative to thermal activation for fabricating low-dimensional carbon-based nanomaterials, offering unique advantages such as non-thermal initiation and high chemoselectivity. Controlling the selectivity and efficiency of on-surface photoreactions remains challenging due to the complex interplay among molecular excitation pathways, substrate properties, and reaction conditions. This review briefly summarizes recent advances in light-driven on-surface synthesis under ultra-high-vacuum conditions. We focus on molecular photoexcitation pathways that can be probed by scanning tunneling microscopy and spectroscopy (STM and STS). Studies of light-driven reactions in three categories are overviewed, i.e., dehalogenative C-C coupling, [2+2] and [4+4] cycloadditions, and photoisomerization. Typical strategies for tuning reactivity are exemplified, including molecular pre-organization via self-assembly, surface passivation, and wavelength/polarization control. The summary of successful case studies may not only facilitate the fundamental understanding of on-surface photochemistry but also inspire the design of functional low-dimensional architectures and light-responsive molecular devices.

## 1. Introduction

On-surface synthesis (OSS) has emerged as a revolutionary bottom–up strategy for the precise fabrication of low-dimensional nanostructures and functional molecules [1,2,3,4,5,6].The advancement of OSS is inherently linked to the evolution of activation methodologies, with thermal annealing, light irradiation, and scanning tunneling microscope (STM) tip-induced excitation being the three dominant strategies driving surface reactions [7]. Among these, thermally activated reactions have long propelled the field, particularly on metal substrates where catalytic properties facilitate key processes such as Ullmann coupling [8,9,10,11,12,13]. However, high temperatures often induce molecular thermal desorption and an increase in surface defects, while the lack of chemical selectivity frequently gives rise to unwanted side reactions [10,14]. Conversely, STM tip-induced excitation enables site-specific initiation at the single-molecule level but suffers from limited scalability for uniform large-area reactions and strong dependence on precursor structures and substrate conductivity [2,15].

These inherent limitations have motivated the exploration of alternative non-thermal strategies, among which light-driven activation stands out as a particularly promising approach [16]. Photochemical activation enables selective excitation of targeted chemical bonds under mild conditions by tuning the wavelength to match characteristic molecular absorptions, thereby avoiding thermal degradation and desorption [17]. Furthermore, it supports highly selective reactions on diverse substrates, including semiconducting and insulating surfaces where thermal or tip-based methods face significant challenges [18,19,20].

Light-induced bond dissociation [10,21,22,23,24] and formation [7,25,26,27,28] have been realized across a variety of on-surface systems, enabling diverse reaction pathways under mild conditions. These photoreactions can be broadly categorized into several types based on the underlying activation mechanisms and structural outcomes. For instance, direct excitation of molecular chromophores has been employed to drive metal-free homocoupling of terminal alkynes on highly oriented pyrolytic graphite (HOPG) surfaces [25], as well as to induce photopolymerization of maleimide monomers into extended covalent fibers on insulating substrates [26]. In parallel, substrate-mediated excitation pathways, particularly on noble metal surfaces [24,29], have enabled visible-light-induced bond cleavage and subsequent coupling reactions, such as the formation of higher acenes from α-bisdiketone precursors [27] or the Ullmann-type coupling of aryl halides under combined photochemical and thermal conditions [22]. These representative examples illustrate the broad applicability of light-driven strategies in constructing low-dimensional nanostructures with atomic precision.

This review aims to systematically summarize the latest advances in light-driven on-surface reactions under ultra-high-vacuum conditions, with an emphasis on elucidating the excitation mechanisms and reaction pathways associated with different photo-driven activation modes. It also explores the influences of key experimental parameters, including surface passivation strategies, light wavelength and polarization tuning, and molecular pre-organization via self-assembly, on the outcomes of on-surface photoreactions. To provide a comprehensive overview, this minireview consists of four main sections. (1) The first section discusses the fundamental mechanisms of on-surface photoexcitation, distinguishing between direct intramolecular excitation and substrate-mediated processes. (2) The second section categorizes key reaction classes enabled by light, including dehalogenative coupling, cycloadditions, and isomerization. (3) The third section outlines strategies for controlling reactivity, with a focus on molecular preorganization, surface passivation, and light parameter engineering. (4) The final section compares thermal and photoinduced synthesis in terms of bond activation, morphological control, and intermediate stabilization. These discussions lay a foundation for developing efficient and selective light-driven synthetic routes toward functional low-dimensional architectures.

## 2. Mechanisms of On-Surface Photo-Excitation

**Figure 1:**
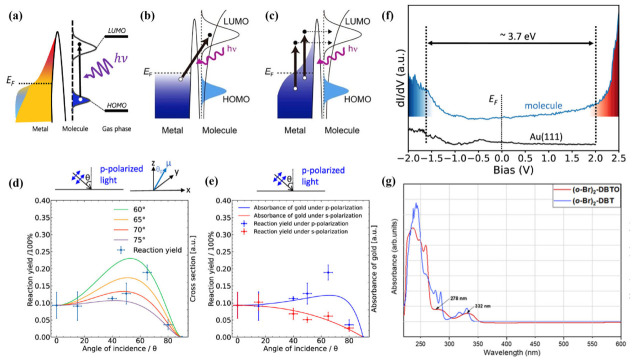
(**a**) Direct excitation: the adsorbates are activated through direct excitation between the HOMO- and LUMO-derived molecular states. The HOMO and LUMO of the molecule in the gas phase are shown for comparison [7]. (**b**) Charge transfer from metal bulk or surface states to unoccupied molecular orbitals. (**c**) Hot carriers from bulk photoabsorption transfer to unoccupied molecular orbitals. Black and white dots denote electrons and holes, respectively [31]. (**d**) Experimentally determined reaction yields under p-polarized light (blue dots) as a function of incidence angle. (**e**) The experimentally determined reaction yields (blue dots and red dots) and the calculated dependences of absorbance of gold on the angle of incidence (bule and red lines) [7]. (**f**) Scanning tunneling spectroscopy (STS) measurements of the 4,4″-Dibromo-p-terphenyl (DBTP) molecule on Au(111). The reference spectrum acquired on the bare surface is displayed in black. The relative frontier molecular orbitals are depicted with fillings in blue for HOMO and red for LUMO [33]. (**g**) The UV-vis optical absorption spectrum of (o-Br)_2_-DBTO (red curve) in dichloromethane (DCM) solution (10^−4^ M) displays two wide absorption bands peaking at 332 and 278 nm, and a main feature in the 260–230 nm range [34].

On-surface photochemical reactions are primarily governed by two core mechanisms [30]: direct intramolecular excitation (Figure 1a) and substrate-mediated excitation (Figure 1b,c), which includes both hot carrier excitation (Figure 1c) and charge transfer from the substrate Fermi level to the molecular LUMO (Figure 1b) [7,31,32]. These two excitation pathways enable precise control over the reaction routes of surface-adsorbed molecules, thereby opening novel avenues for the design and development of on-surface chemical reactions. This section details each mechanism and discusses the regulatory role of the substrate in steering photoreactivity.

### 2.1. Direct Intramolecular Excitation

Direct intramolecular excitation [4] represents a fundamental photochemical pathway in which an adsorbed molecule absorbs a photon and undergoes an electronic transition from its ground state to an excited state, forming an exciton that remains confined within the molecule. It is noteworthy that this excitation process is entirely confined within the molecule, and the molecular charge state remains unchanged following photon absorption. A prerequisite for this process is that the indirect photon energy matches the characteristic electronic transition of the target molecule, a condition known as resonance excitation.

A representative example of this mechanism is the photoinduced deoxygenation of 4,6-dibromodibenzo[b,d]thiophene 5-oxide ((o-Br)_2_-DBTO) on NaCl thin films grown on Au(111), as reported by Nony et al. [34] In this system, the insulating NaCl layer electronically decouples the molecules from the metallic substrate, preserving their intrinsic photophysical properties. The Ultraviolet–Visible (UV–vis) absorption spectrum of (o-Br)_2_-DBTO in dichloromethane exhibits characteristic bands centered at approximately 280 nm and 330 nm, establishing the resonance condition for photoexcitation (Figure 1f). Upon irradiation with Ultraviolet A (UVA 280–365 nm) light in the range of 280 to 365 nm, the molecule undergoes direct excitation from the ground state (S_0_) to the lowest singlet excited state (S_1_), leading to efficient deoxygenation. The choice of the NaCl thin film is critical, as it ensures that the absorbed photon energy is fully utilized for intramolecular electron transitions rather than being dissipated through substrate–molecule interaction [35].

Beyond single-molecule deoxygenation reactions, direct intramolecular excitation has also been successfully extended to on-surface photopolymerization for fabricating ordered nanostructures. For instance, Grossmann et al. [36] demonstrated the synthesis of mesoscale-ordered porous two-dimensional polymers (2DPs) via topochemical photopolymerization of fluorinated anthracene triptycene (fantrip) monomers on alkane-passivated graphite surfaces. Here, the passivation layer plays a dual role: it electronically decouples the monomers from the conductive graphite support, and it enforces a specific molecular orientation that satisfies the geometric requirements for [4+4] cycloaddition. The reaction selectivity is further governed by the alignment between the incident light polarization and the monomer’s transition dipole moment (TDM). Since the TDM of fantrip is parallel to its molecular symmetry axis, only p-polarized light in the 395–405 nm range efficiently excite the π electrons of the anthracene units to the S_1_ state, triggering covalent crosslinking while preserving long-range order. Notably, the polymerization degree is independent of the excitation source (Light Amplification by Stimulated Emission of Radiation, laser or LED, Light-Emitting Diode) as long as the photo energy exceeds the S_1_ state energy, confirming that the reaction proceeds via direct photoexcitation of the monomers rather than through substrate-mediated processes [36].

Similar strategies have been employed in other systems where direct intramolecular excitation enables precise control over reaction pathways. For example, the photopolymerization of diacetylene derivatives on insulating or passivated surfaces relies on the same principle: the substrate decouples the organic layer, allowing the intrinsic photophysics of the monomers to dictate the reaction outcome [37,38,39]. Collectively, these studies underscore that direct intramolecular excitation offers a versatile platform for achieving chemoselective and spatially controlled on-surface reactions, provided that the molecular excited states are effectively shielded from substrate-induced quenching.

### 2.2. Substrate-Mediated Excitation

In contrast to direct intramolecular excitation, substrate-mediated excitation relies on photon absorption by the underlying substrate rather than by the adsorbate itself. This indirect pathway is particularly prevalent on metal surfaces, where the high density of electronic states near the Fermi level enables efficient light–matter coupling. As illustrated in Figure 1b, substrate-mediated excitation encompasses two key sub-pathways: hot carrier excitation and charge transfer. Although both mechanisms ultimately deliver energy to the adsorbed molecule, they differ fundamentally in their physical origin, the required optical excitation conditions, and the transient molecular charge states [7,31,40].

Hot carrier excitation occurs when the metal substrate absorbs incident photons, generating non-equilibrium electrons and holes [41,42,43]. These hot carriers can subsequently tunnel across the molecule–substrate interface and inject into the frontier molecular orbitals (e.g., the lowest unoccupied molecular orbital, LUMO) of the adsorbate, thereby promoting the molecule to an excited state and triggering bond dissociation. A representative demonstration of hot carrier-mediated chemistry was reported by Sun et al. [7] using the photoinduced debromination and coupling of 4,4″-dibromo-p-terphenyl (DBTP) on Au(111) under 405 nm linearly polarized light. The study clarified that the reaction yield exhibits a clear dependence on the polarization state of the light and incidence angle (Figure 1c,d). Systematic measurements reveal that the maximum yield occurs at an incidence angle of approximately 66°, a value that precisely aligns with the calculated absorbance profile of the Au substrate rather than the orientation of the molecular transition dipole moment. This alignment provides definitive evidence for a hot-electron-driven process. Additionally, the non-thermal nature of this excitation often results in reaction products, such as short-chain oligomers, that are morphologically distinct from those produced via thermal annealing.

The charge transfer excitation involves direct electron transfer from the Fermi level of substrate to the LUMO of the adsorbate upon photon absorption. Unlike hot carrier excitation, which involves non-equilibrium carriers generated within the substrate, charge transfer excitation can occur when the photon energy matches the energy difference between the Fermi level of the metal and the LUMO of the molecule. This mechanism is typically operative at longer wavelengths, including the visible and near-infrared regions, and likewise results in the formation of a transient anionic state on the molecule. Building on their earlier work, Sun et al. [33] further demonstrated that the same DBTP/Au(111) system can be activated using near-infrared light (700–750 nm). Scanning tunneling spectroscopy (STS) measurements (Figure 1e) revealed that the energy difference between the Au(111) Fermi level and the LUMO of DBTP is approximately 1.8 eV, matching the incident photon energy with high precision. This energy alignment facilitates a direct electron transfer from the substrate to the molecule. In contrast, the significantly larger highest occupied molecular orbital (HOMO)-LUMO gap of 3.7 eV precludes direct intramolecular excitation under these irradiation conditions. Collectively, these complementary studies establish a robust experimental framework for distinguishing hot carrier excitation from charge transfer mechanisms on metal surfaces [43,44].

### 2.3. Substrate Effects in On-Surface Photochemistry

The electronic and structural properties of substrates play a decisive role in determining both the excitation pathways and the overall efficiency of on-surface photochemical reactions. As discussed in the previous sections, the two fundamental excitation mechanisms, namely direct intramolecular excitation and substrate-mediated excitation, are each favored by different substrate characteristics. Accordingly, substrates can be broadly categorized into metallic, insulating, and semiconducting types, each imposing a unique regulatory influence on reaction pathways, selectivity, and yield.

Metallic Substrates: noble metal substrates [45] (e.g., Au, Ag, Cu) exert a dual influence on on-surface photochemical processes. On one hand, they serve as the primary source of hot carriers (hot electrons and holes) upon light absorption, enabling substrate-mediated excitation pathways that extend molecular photoreactivity into the visible and near-infrared regimes, as exemplified by the hot carrier and charge transfer mechanisms discussed in Section 2.2 [7,33]. On the other hand, the strong electronic coupling between metallic surfaces and adsorbed molecules facilitates ultrafast quenching of molecular excited states. This quenching dissipates photon energy through substrate–molecule electronic interactions, impairs the reactivity of photoexcited molecules, and ultimately restricts the overall efficiency of photochemical reactions.

Insulating substrates offer a contrasting environment by effectively decoupling adsorbed molecules from any underlying conductive support. Materials such as NaCl thin films, calcite, and alkane-passivated graphene minimize electronic interactions between the molecule and the substrate, thereby eliminating the excited-state quenching inherent to metals [34,36]. This decoupling preserves the intrinsic photophysical properties of molecules, allowing direct intramolecular excitation to proceed as the dominant reaction mechanism. Consequently, photon energy is fully utilized for targeted electronic transitions, enabling highly selective photoreactions that closely mirror the solution-phase photochemistry of the isolated molecules.

**Figure 2:**
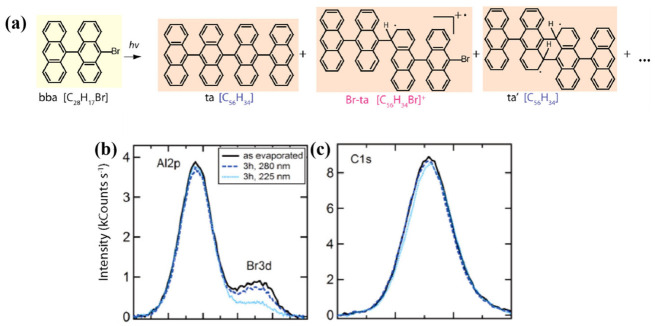
(**a**) Upon UV irradiation in nanometer-thin films under UHV conditions, 10-bromo-9,9′-bianthryl (bba, 432 g·mol^−1^) can react producing 9,10-tetraanthrylene (ta, 706 g·mol^−1^), Br-ta (786 g·mol^−1^) or the ta isomer (ta′), among others. (**b**,**c**) Br3d and C1s spectra of the bba multilayer film [47].

Wide-bandgap insulating surfaces, such as KCl [46], SiO_2_ [47], h-BN [48], and calcite [49], naturally decouple molecules from underlying conductive supports, thereby suppressing substrate-mediated processes [47,50]. This physical decoupling creates an environment where direct molecular excitation can dominate. For example, on the h-BN(0001) surface, the photopolymerization time of 10,12-Pentacosadiynoic acid (PCDA) molecules is significantly reduced by two orders of magnitude (to 10 s) compared to that on graphite, attributed to the wide bandgap (5.79 eV) of h-BN, which suppresses the relaxation channels of photogenerated intermediates [51]. Similarly, on the KCl(100) surface, maleimide molecules achieve directional growth via specific interactions between the oxygen atoms and potassium cations, enabling UV-triggered free radical chain polymerization into micrometer-long covalent fibers [26]. On insulating substrates like mica or SiO_2_/Si, the photochemical cleavage of C–Br bonds in 10-bromo-9,9′-bianthracene (bba) multilayers proceeds efficiently (Figure 2a). XPS confirms that the carbon backbone is retained (Figure 2b,c) and can subsequently form higher molecular weight coupling products upon annealing, a behavior not observed in the absence of UV pre-exposure [47].

Semiconducting substrates occupy an intermediate position, combining advantageous features of both metals and insulators. Their intrinsic bandgap enables wavelength-selective photocatalysis, while their superior charge separation performance mitigates electron–hole recombination and prolongs the lifetime of molecular excited states relative to metallic substrates [50]. These characteristics can further facilitate efficient direct photochemical processes, offering additional flexibility in reaction design.

Semiconductors offer an intermediate passivation strategy [52], combining decoupling with the ability to harvest light. Their intrinsic bandgap allows for wavelength-selective excitation, while their charge separation properties prolong excited-state lifetimes [50]. On SnSe, for instance, the selective photodissociation of tetraphenylphthalic anhydride (TPPA) over a structurally similar analogue (BPA) is dictated by the dissociative n → π* nature of its S_1_ state, as revealed by density functional theory (DFT) and Algebraic-Diagrammatic Construction to second order (ADC (2)) calculations. The surface itself remains passive, enabling the reaction to be controlled by the molecular electronic structure rather than by hot carriers [50].

Overall, the electronic and structural characteristics of substrates are core determinants of the excitation mechanism and reaction outcome in on-surface photochemistry. Strategic selection of substrate materials, whether to harness hot carrier generation on metals, to preserve molecular excited states on insulators, or to exploit wavelength-selective photocatalysis on semiconductors, provides a powerful lever for designing efficient and selective photochemical systems. Manipulating substrate–molecule interactions thus emerges as a critical strategy for achieving precise control in light-driven on-surface synthesis.

## 3. Light-Driven Reaction Types

### 3.1. Dehalogenative C-C Coupling

Light-induced dehalogenation [53,54,55] has emerged as a powerful strategy for on-surface C-C bond formation, enabling highly selective coupling under mild conditions that circumvent the limitations of conventional thermal Ullmann reactions. Compared to thermal activation, which often requires elevated temperatures that can trigger side reactions such as C-H activation or molecular desorption, photochemical excitation enables cleavage of C-X bonds (X = Br, Cl, I) at low temperatures [14], thereby offering enhanced control over reaction pathways and product selectivity [19].

A range of studies have demonstrated the effectiveness of this approach across different metal substrates. Langlais et al. successfully lowered the reaction temperatures for each stage of the Ullmann coupling of 4,4″-dibromo-p-terphenyl (DBTP) on Ag(100) by 50 K using a combination of UV–visible–near-infrared light and thermal treatment. They proposed that hot electrons generated by surface plasmon excitation play a Key role in C-Br bond cleavage [31]. Similarly, Zuzak et al. utilized light-induced dechlorination reactions on the Au(111) surface to activate aryl chlorides under conditions ranging from room temperature to 365 K, providing an environmentally friendly reaction pathway for chlorinated aromatics with high C-Cl bond energies [21].

**Figure 3:**
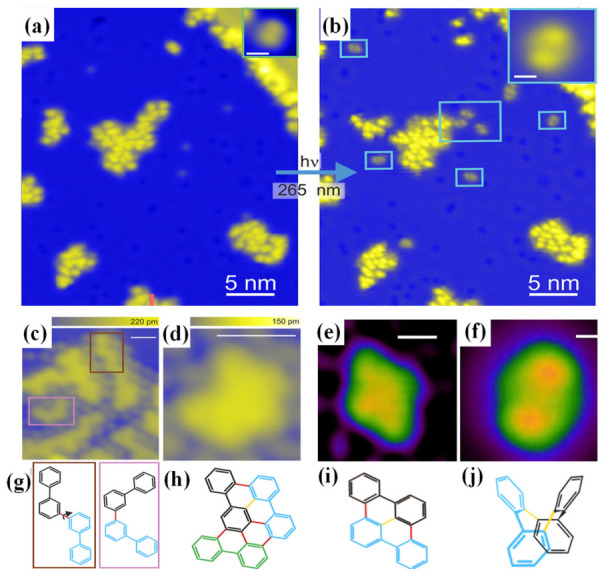
Photolysis activation vs. thermal activation of the Ullmann coupling: (**a**) STM image after adsorption at 150 K; (**b**) STM image after 18 h of 265 nm light exposure; (**c**,**d**) STM images of a few products after annealing at 520 K; (**e**) STM image of the main product obtained after thermal activation at 520 K; (**f**) STM image of the sole product obtained through photolysis activation; (**g**–**j**) corresponding chemical structures derived from the STM images. The newly formed bonds are highlighted in yellow (Ullmann coupling) and red (C-H activation) Pink and brown boxes in (**c**) mark a zigzag and C-shaped structure, respectively. The arrow in (**g**) marks the rotation that converts a zigzag to a C-shaped structure [12].

In terms of selectivity control, photolysis activation demonstrates a significant advantage over thermal methods. For example, in the reaction of 2,2′-dibromobiphenyl on Cu(111), as shown in Figure 3, thermal activation produces diverse products (diamond-shaped, chain-like, and arrow-shaped), whereas photolysis activation generates only a single product, tetra-phenyl. DFT calculations show that light excitation elevates the system to an excited-state potential energy surface, activating reaction channels that thermal activation cannot reach [12].

**Figure 4:**
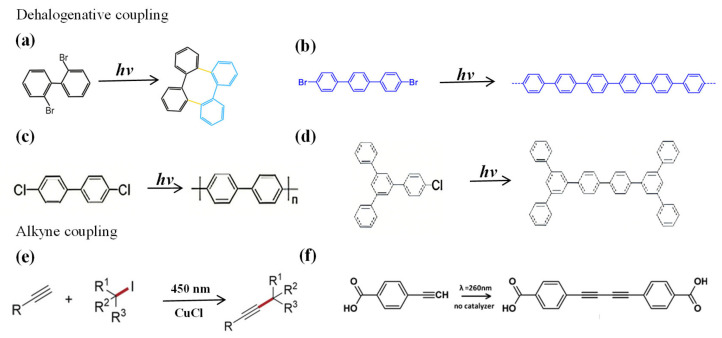
Schematic of selected reactions commonly used in surface synthesis, divided into dehalogenative coupling ((**a**) [12], (**b**) [33], (**c**,**d**) [14]) and alkyne coupling ((**e**) [56], (**f**) [25]).

**Figure 5:**
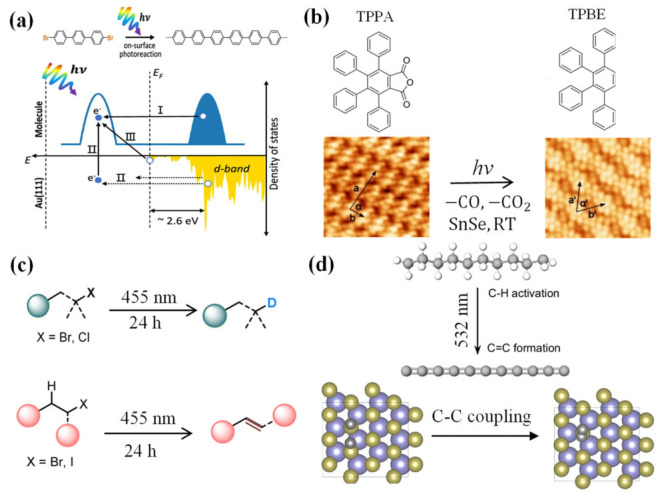
(**a**) Three mechanisms of light-induced dehalogenation coupling of DBTP precursors on Au(111). I represents the direct excitation process; II represents the indirect hot electron mechanism; III represents the charge transfer process from the Fermi level of the metal substrate to the lowest unoccupied molecular orbital [33]; (**b**) Photolysis of the anhydride group of derivatives TPPA and BPA on SnSe: STM images before and after 280 nm light exposure at room temperature [50]; (**c**) Selective deuteration (**top**) and elimination reactions (**bottom**) of alkyl halides [57]; (**d**) Photochemical processes for C-H bond activation and C=C bond formation (**top**), and DFT simulation of the C-C coupling process on the WSe_2_ surface (**bottom**) [58].

The mechanism of light-induced dehalogenation reactions exhibits significant wavelength dependence. On the Au(111) surface, the efficiency of photodebromination coupling of DBTP (Figure 4b) follows the order of 313 nm > 405 nm > 700–750 nm (Figure 5b), corresponding to direct molecular excitation, hot electron mechanisms, and charge transfer mechanisms, respectively (Figure 5a). STS measurements reveal that the HOMO–LUMO gap of DBTP is approximately 3.7 eV, which closely matches the photon energy of 313 nm (4.0 eV) [19].

Beyond noble metal surfaces, light-induced dehalogenation reactions can also be extended to semiconductor surfaces (Figure 5b). Studies have found that on the SnSe semiconductor surface, selective cleavage of the anhydride group and subsequent coupling can be achieved through light exposure, providing a new pathway for constructing covalent nanostructures on non-metallic substrates [50]. Moreover, light excitation strategies have also been successfully applied to the selective deuteration and elimination of alkyl halides [57] (Figure 5c), as well as C–H activation and coupling in alkanes [58] (Figure 5d), demonstrating its broad potential in precise synthesis and molecular modification.

In addition to expanding the scope of Ullmann-type couplings, light excitation offers new strategies for constructing structurally precise conjugated systems [59]. For example, through surface-guided photochemical tetramerization, cycloheptatriene can be synthesized in a single step on the Cu(100) surface, highlighting the unique role of the surface in controlling reaction pathways and stereoselectivity [60]. Collectively, these studies demonstrate that the mechanisms of light-induced dehalogenation and C–C coupling reactions encompass multiple pathways, including molecular excitation, hot electron injection, and charge transfer. In the future, strategies combining photochemistry and surface science are expected to play a greater role in the precise synthesis of complex nanostructures such as graphene nanoribbons and dendritic molecules [19,61].

### 3.2. Cycloadditions ([2+2] and [4+4])

**Figure 6:**
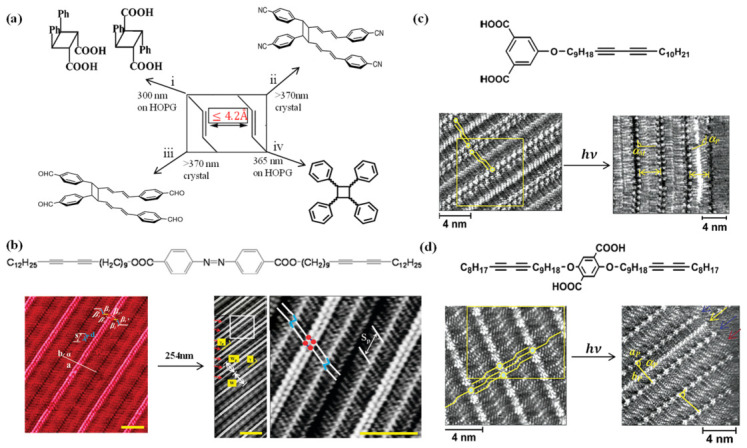
(**a**) Chemical schematic of the [2+2] photochemical cycloaddition reaction, illustrating the optimal distance for the reaction evolution according to topochemical postulates. (i) Photoreaction pathway of AOCA precursor on highly oriented pyrolytic graphite (HOPG) [46]; (ii) and (iii) Crystalline-state [2+2] photodimerization of 4,4′-dicyanobenzene substituted (E,E,E)-DPH4 and 4,4′-formyl substituted (E,E,E)-DPH4 at the terminal double bond of the triene [67]; (iv) Photodimerization of porphycene on HOPG under UV light [66]. (**b**) Chemical structure of Azo-DA diacetylene azo derivative, with high-resolution STM images showing polymerization on HOPG under UV illumination. The diacetylene units are indicated with red arrows. S and S_p_ are the distance between every fifth alkyl chain. The unit cell parameters are a = (8.0 ± 0.1) nm, b = (0.5 ± 0.05) nm, and α = (87 ± 1)°) and the angles are β1 or β1′ = 115 ± 2°, β2 or β2′ = 109 ± 2° and β3 = 129 ± 2° and β3′ = 125 ± 3°. The red arrows indicate bright lines that were not observed before irradiation (Wp = 7.2 ± 0.2 nm, W = 7.9 ± 0.1 nm) [39]. (**c**) Chemical structure of ISA-DIA, showing high-resolution STM images of polymerization on graphite under UV illumination, with corresponding angles of alkyl chains in the polymerized and unpolymerized diacetylene arrays relative to the direction of the polymer film. Arrows in the figure indicate the layer width. Four molecules are drawn in yellow. α_P_ and α_M_ are the alkyl chain angles of the polymerized and nonpolymerized diacetylene arrays, respectively. [38]. (**d**) Chemical structure of TTA-DIA, showing high-resolution STM images of polymerization on graphite under UV illumination. The polymerized array has unit cell parameters a_P_, b_P_, and α_P_. In (**c**,**d**), blue, red, and yellow arrows point to the ISA and TTA groups, unpolymerized diacetylene arrays, and polymerized diacetylene arrays, respectively [38].

Photochemical cycloaddition reactions [62,63] serve as an important strategy for surface-limited photopolymerization, enabling the construction of covalent bonds through [2+2] and [4+4] cycloadditions [64,65]. These reactions allow for the precise formation of one-dimensional and two-dimensional polymeric nanostructures under mild conditions. According to topochemical principles, photochemical cycloaddition reactions require the double bonds of adjacent reaction units, whether olefins or alkynes, to be aligned in parallel with a distance ≤0.42 nm (Schmidt’s rule, Figure 6a). This geometric constraint must be satisfied through molecular design in surface self-assembled monolayers [19,50].

**[2+2] Photocycloaddition**. A variety of molecular systems have been explored to meet these topochemical requirements. On the HOPG surface, porphycene undergoes light-induced isomerization to the cis configuration under 365 nm UV illumination, aligning adjacent double bonds for [2+2] photodimerization reaction to form a cyclobutane product (Figure 6a, route iv). STM images clearly show that one row of molecules in the dimer displays a darker four-lobed feature, corresponding to the cyclobutane benzene rings, while the biphenyl core of the other row undergoes a conformation change from 200°to 144°, revealing the decisive influence of surface topology on reaction stereoselectivity [66].

For diacetylene compounds, the 1,4-photopolymerization reaction requires adjacent diacetylene units to be spaced approximately 0.5 nm apart with an angle of about 45° relative to the stacking axis. These conditions are satisfied in the self-assembly structures of the isophthalic acid derivative (ISA-DIA) and the terephthalic acid derivative (TTA-DIA) molecules on the graphite surface. Upon UV irradiation, a conjugated polybutadiyne backbone is formed. STM images acquired in constant height mode reveal that the polymerized main chain exhibits brighter contrast, indicating its partial lifting from the surface. Concurrently, the orientation angle of the alkyl chains undergoes a significant change [37,38]. Similarly, Azobenzene-Diacetylene (Azo-DA) molecules, connected by long alkyl ester linkers, form a layered structure on HOPG with diacetylene spacing of 0.5 nm and an angle of 47°. Exposure to 254 nm UV light yields a zigzag polybutadiyne backbone (Figure 6b), with layer width contracting from 7.9 nm to 7.2 nm while the azo-benzene units retain their trans configuration [39]. Additionally, 4-pentoxy cinnamic acid (AOCA) forms self-assembled structures on HOPG that satisfy topochemical polymerization requirements, undergoing photodimerization after UV exposure (Figure 6a, route i), demonstrating the applicability of carboxyl hydrogen bonding systems in surface photopolymerization [19,50].

**Figure 7:**
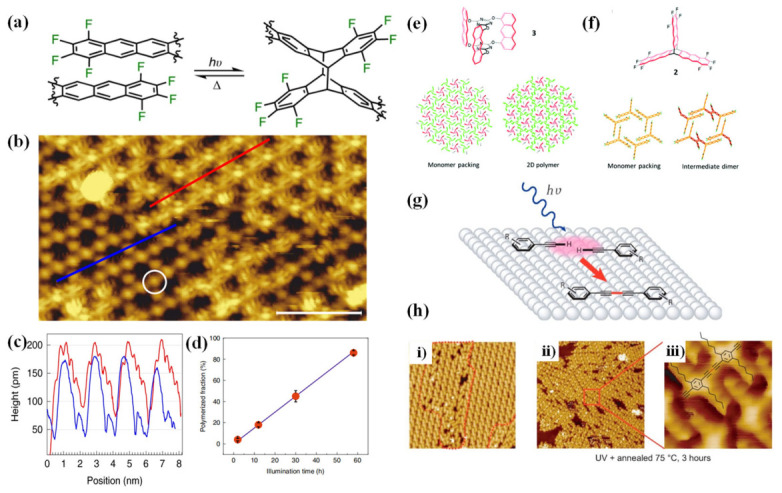
(**a**) Chemical schematic of the [4+4] photochemical cycloaddition reaction of Fantrip; (**b**) Hexagonal Fantrip monolayer structure on alkane-passivated graphite surface; (**c**) Line profiles as indicated in (b). For the brighter region (red), the averaged interpore distance is 2.02  ±  0.05 nm, notably larger than the 1.88  ±  0.05 nm for the darker region (blue); (**d**) Relationship between polymerization fraction and light exposure time [36]; (**e**) Molecular structure of the monomer utilized to synthesize 2DPs (monomer 3), and its single-crystal structures in the monomer, dimer intermediate, and corresponding two-dimensional polymer 2DP2 [69]; (**f**) Chemical structure of the monomer fantrip (monomer 2), its stacking arrangement in a single crystal, and the corresponding two-dimensional polymer 2DP3 structure in the crystal [70]; (**g**) Schematic of the light-induced surface laser polymerization reaction; (**h**) (i) High-resolution STM image of the polymerized product after UV exposure; (ii,iii) STM images of the aromatic alkyne oligomers on thermally treated Ag(111) [28].

**[4+4] Photocycloaddition**. The [4+4] photochemical cycloaddition demonstrates unique advantages in the synthesis of two-dimensional polymeric nanostructures. The fluorinated anthracene triptycene (fantrip) molecule forms a hexagonal porous monolayer on alkane-passivated graphite through the upright arrangement of its three anthracene units (Figure 7b). Adjacent anthracene units are spaced approximately 0.4 nm, satisfying the geometric requirements for topochemical [4+4] cycloaddition. Upon exposure to 405 nm UV light, covalent cross-linking occurs (Figure 7a), yielding a two-dimensional polymer. STM images show that the lattice constant contracts from 2.05 nm to 1.88 nm (Figure 7b,c), with polymerized regions appearing darker. The polymerization fraction increases linearly with exposure time, reaching up to 90% (Figure 7d), a behavior attributed to disruption of the anthracene aromatic system that widens the energy gap [36].

The polymerization mechanism can differ markedly between bulk crystals and surface monolayers. In the single crystals, fantrip (monomer 2) [68] forms stacked structures with vertically aligned anthracene pairs (Figure 7f). X-ray diffraction indicates a polymerization mechanism intermediate between random and self-suppressing, where the distance between unreacted anthracene pairs increases progressively, relieving strain and preventing crystal fragmentation. In contrast, on surface monolayers, the monomer utilized to synthesize 2DPs (monomer 3) [68] follows a self-accelerating mechanism, with polymerization preferentially propagating from the edges of existing polymer domains,akin to chain-growth polymerization (Figure 7e).

**Wavelength control and reaction mechanisms**. The mechanistic analysis and wavelength control of photochemical cycloaddition reactions provide a foundation for their application in on-surface synthesis. On Ag(111), UV light (375 nm) efficiently induces Glaser coupling of terminal alkynes (Figure 7h), predominantly producing diacetylene-linked dimers (87.4%) (Figure 7(h-i)). In contrast, thermal activation yields long-chain polymers with pentamers (Figure 7(h-ii,h-iii)) but with reduced order, highlighting the ability of light to achieve spatiotemporally controlled C-C bond formation while maintaining self-assembled structure [28]. On Au(111), light-induced dechlorination coupling under 405 nm or 254 nm light exposure activates C-Cl bonds at room temperature to 365 K, forming short-chain oligomers, whereas thermal activation requires approximately 573 K, effectively suppressing diffusion-induced side reactions [14].

Wavelength-dependent studies further reveal the complexity of photochemical cycloadditions. For the visible light-induced [2+2] cycloaddition of styrene-phenanthrene, wavelength-dependent photon efficiency analysis (WPEA) identified optimal wavelength of 435 nm for cycloaddition and 330 nm for ring cleavage, which deviate from the UV/vis absorption maximum (around 375 nm), underscoring the intricate relationship between photochemical efficiency and absorbance [71]. Meanwhile, photoinitiated free-radical chain polymerization of maleimide on KCl insulating surfaces shows that UV exposure enhances polymerization efficiency by two orders of magnitude, producing covalent fibers up to 1 μm in length. Nudged elastic band (NEB) calculations confirm a chain-growth mechanism, where a photogenerated triplet diradical reacts with a second molecule to form a diradical dimer, with an energy 0.40 eV lower than that of the adsorbed reactant [26]. These studies systematically reveal the synergistic effects of molecular preorganization, surface topology, and excited-state dynamics in surface-limited photochemical cycloadditions, providing both theoretical guidance and experimental paradigms for the development of structurally precise conjugated polymer nanomaterials.

### 3.3. Isomerization

Photochemical isomerization reactions [72,73,74] serve as a core strategy for constructing light-responsive molecular switches, enabling reversible regulation of self-assembled structures through light-induced molecular configuration changes. These reactions exhibit distinct mechanistic features in surface-confined systems, which are primarily governed by the electronic properties of the underlying substrate [19].

On metal surfaces, photochemical isomerization proceeds via the surface photoinduced electron transfer (S-PET) mechanism. In this pathway, photons absorbed by the substrate generate hot electrons that are injected into the unoccupied orbitals of the adsorbed molecule, forming anionic excited states that subsequently trigger configuration changes. A representative example is the cis-trans isomerization of porphycene molecules on Cu(111), where the reaction yield exhibits a sharp increase around a photon energy of approximately 2 eV, closely matching the d-band edge energy of copper and confirming the dominant role of the S-PET mechanism [19,21].

On insulating surfaces, photochemical isomerization primarily proceeds through direct molecular excitation. Upon photon absorption, the molecule transitions to an excited state and relaxes to a reactive excited state via internal conversion or intersystem crossing [39,66]. Surface adsorption can significantly influence photoisomerization behavior through spatial confinement effects and electronic structure reorganization, providing important design principles for light-responsive molecular switches [19].

Azo-benzene and stilbene derivatives serve as model systems for understanding surface-confined photoisomerization. The Azo-DA (azobenzene-diacetylene) molecules, containing both azo-benzene and diacetylene photoactive groups, self-assemble into highly ordered layered structures on HOPG. The trans-azo-benzene units appear as two separated bright spots in STM images (Figure 6b). After exposure to 365 nm UV light, part of the trans-azo-benzene undergoes isomerization to the cis configuration, presenting continuous bright line features. The molecular length shortens from 1.1 nm to 0.9 nm, and the apparent height increases by approximately 0.14 nm. Further exposure to 435 nm visible light reversibly restores the cis configuration to the trans configuration, returning the self-assembly to its original state. Notably, this reversible photochemical isomerization is also observed in the polymerized Azo-poly (DA), indicating that the conjugated diacetylene backbone does not affect the light-induced isomerization activity of the azo-benzene units [39].

**Figure 8:**
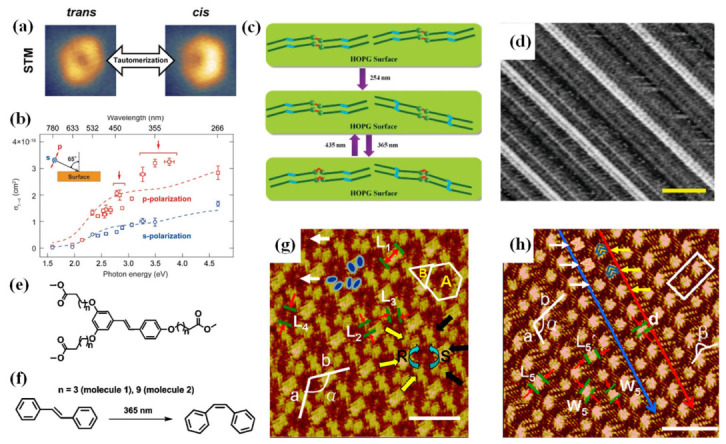
(**a**,**b**) STM images of trans- and cis-configurations of porphycene molecules on Cu(111), with wavelength (photon energy) dependence measured using p-polarized (red squares) and s-polarized (blue squares) light [35]; (**c**,**d**) Schematic model and STM images of the reversible photochemical isomerization between the trans and cis isomers of Azo-DA and Azo-poly(DA) [39]; (**e**) Chemical structures of two porphycene derivatives (molecule 1 and molecule 2); (**f**) Reaction mechanism for cis-trans isomerization of molecule 1; (**g**,**h**) STM images of the adsorption conformations of molecule 1 and molecule 2 after UV light exposure. Two types of cavities are marked A and B. The R-configuration is indicated by the yellow arrow, and the S-configuration by the black arrow. The unit cell parameters are a = 3.9 ± 0.1 nm, b = 4.0 ± 0.1 nm, and α = 121 ± 2° [66].

The study of stilbene derivatives further reveals the significant influence of molecular structural modifications on photoisomerization behavior. As shown in Figure 8e, molecule 1 (with a shorter alkoxy chain) undergoes cis-trans isomerization upon exposure to 365 nm UV light for 10 min (Figure 8d), with the self-assembled structure transitioning from a layered arrangement to a Kagome network with hexagonal A-type and triangular B-type cavities (Figure 8e). The cis-stilbene core adopts a “V” shape, the molecular length shortens from 1.3 nm to 1.1 nm, and each A-type cavity is formed by six molecules in a chiral configuration. In contrast, molecule 2 (with a longer alkoxy chain) forms a layered structure composed of dimers after 5 min of the same UV light exposure (Figure 8f). Within each dimer, two cis-molecules adopt two distinct conformations, namely “head-to-head” and “head-to-tail” [33]. UV-vis spectra confirm the presence of cis-isomers in solution, with the absorption peak shifting from 323 nm to 273–274 nm, consistent with STM observations [39].

These studies provide the first real-time observation of surface photochemical isomerization and its dynamic control over self-assembly structures, offering experimental evidence and theoretical guidance for the development of multifunctional light-responsive surface nanostructures. From a mechanistic perspective, surface-confined photoisomerization can be driven by either direct molecular excitation (on insulating substrates) or substrate-mediated electron transfer (on metals), with the dominant pathway determined by the electronic coupling between the molecule and the underlying support.

## 4. Strategies for Controlling Reactivity

The efficiency and selectivity of surface-confined photochemical reactions depend critically on the precise control of reaction pathways [75]. Unlike traditional thermally activated processes, light-induced reactions involve the generation and evolution of excited states, and their outcomes are governed not only by the intrinsic molecular structure but also by a combination of factors, including the arrangement of molecules on the surface, the electronic properties of the substrate, and the wavelength and polarization of the incident light. Building on the mechanistic insights discussed in Section 2 and the reaction classes outlined in Section 3, this section will systematically addresses three key control strategies, including molecular self-assembly and preorganization, surface passivation, and wavelength and polarization tuning.

### 4.1. Molecular Assembly and Preorganization

Molecular preorganization on surfaces is the primary prerequisite for determining whether a photochemical reaction can occur and what selectivity it will exhibit [18]. According to topochemical principles, photochemical cycloaddition reactions require the reaction units of adjacent molecules to be aligned in parallel with a spacing of ≤0.42 nm (Figure 6a) [19,50]. For diacetylene compounds, the 1,4-photopolymerization further demands an inter-diacetylene distance of approximately 0.5 nm and an angle of approximately 45° relative to the stacking axis.

These geometric constraints are typically achieved through rational molecular design and self-assembly. On the HOPG surface, ISA-DIA and TTA-DIA molecules form highly ordered layered structures through hydrogen bonding between carboxyl groups and interpenetration of alkyl chains [39]. The stacking distance and angle of the diacetylene units (0.48 nm/50° and 0.485 nm/47°) closely match the optimal parameters for topochemical photopolymerization. Upon UV exposure, a polybutadiyne backbone forms, and STM images show that the polymerized main chain exhibits a brighter contrast under constant height mode, indicating its partial lifting from the surface [37,38]. Similarly, Azo-DA molecules, linked by long alkyl chains, form a layered structure on HOPG surface with a diacetylene spacing of 0.5 nm and an angle of 47°, yielding a zigzag-arranged polybutadiyne backbone after irradiation (Figure 6b) [39]. For [4+4] cycloadditions, Fantrip molecules on alkane-passivated graphite surface form a hexagonal porous monolayer through the upright arrangement of three anthracene units, with as inter-anthracene distance of approximately 0.4 nm that satisfies the geometric requirements for Schmidt’s topochemical photopolymerization. Upon 405 nm UV illumination, covalent cross-linking yields a two-dimensional polymer [36,68].

**Figure 9:**
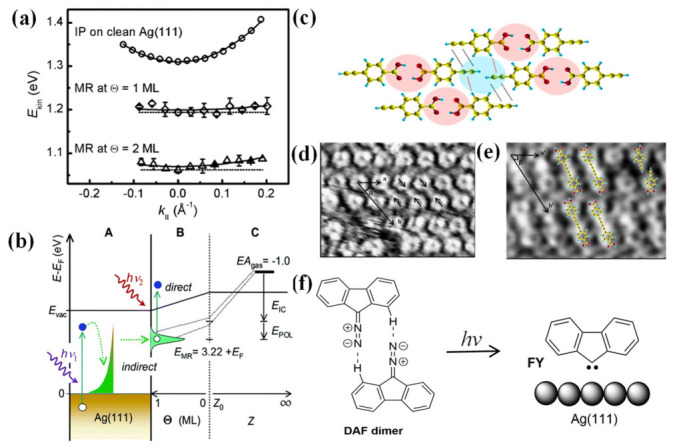
(**a**) Electronic energy dispersion of the intermediate states obtained through angle-resolved 2PPE spectroscopy at different specified coverages; (**b**) Schematic diagram of the hot electron-mediated mechanism generated by light excitation. In column A, a cascade of hot electrons generated by the pump photon hν_1_ scatter into the anionic molecular resonance (MR) state (“indirect” excitation). Before undergoing relaxation, the electron in the transient anion is ejected by the probe photon hν_2_ (“direct” transition ofσ-symmetry, column B). In column C, the energetics of the adsorbate system is shown as a crude function of the distance Z from the substrate [76]; (**c**) Suggested ball-and-stick model of the supramolecular interaction network of PEBA; (**d**) High-resolution image of the ordered hydrogen-bonded dimers; (**e**) High-resolution STM image of photoreaction products and unreacted PEBA monomers. Two collinear hydrogen-bonded carboxylic groups (highlighted in red) and the terminal alkynes (highlighted in blue) [25]; (**f**) Formation of DAF dimers on Ag(111) to create the FY structure [21].

Beyond geometric constraints, precise control of coverage and temperature is essential for achieving molecular preorganization. On Cu(111) surface, deposition of 2,2′-dibromobiphenyl molecules at 150 K provides sufficient surface mobility for the molecules to form adjacent clusters while avoiding thermally induced C-Br bond cleavage, as confirmed by X-ray Photoelectron Spectroscopy (XPS) analysis (Br3p3/2 peak at 184.0 eV). This preorganization lays the structural foundation for subsequent photolysis-activated bimolecular reactions [12]. On Au(111) surface, controlling the coverage of DBTP molecules enables the selective formation of either windmill-shaped or herringbone self-assembled structures. DFT calculations indicate comparable binding energy (approximately 0.18 eV per molecule) with coverage being the only factor driving the structural transition. Light exposure experiments show that the herringbone structure, with higher coverage and more compact molecular arrangement, is more favorable for the light-induced dehalogenation coupling reaction [7,33]. On Ag(111) surface, coverage-dependent two-photon photoelectron spectroscopy (2PPE) reveals that increasing phenol coverage to monolayer induces a molecular resonance state at 3.22 eV above the Fermi level, with an effective mass of (15 ± 10) me (Figure 9b), confirming that excited electrons are mainly localized on the adsorbed molecules [76].

Molecular structure design also plays a decisive role in self-assembly preorganization. A representative example is 4-ethynylbenzoic acid (PEBA) On HOPG, which employs orthogonal supramolecular interactions. Carboxyl groups form collinear hydrogen-bonded dimers, while adjacent ethynyl moieties engage in C≡C-H⋯C≡C type double hydrogen bonds that align them in parallel with a distance of 0.91 nm (Figure 9c,d). This satisfies the geometric requirements for topochemical photochemical reactions. The importance of such designed preorganization is underscored by control experiments, where co-adsorption of terephthalic acid disrupts the ordered network and drastically reduces the photochemical coupling yield to below 10% (Figure 9e) [25].

Similar design principles have been extended to other molecular systems to create preorganized architectures amenable to photochemical activation. For instance, bifunctional porphyrin on Au(111) exploit pyridyl–copper coordination to form one-dimensional supramolecular chains. After annealing at 180 °C, these chains reorganize into double-row and triple-row structures with a well-defined inter-molecular spacing of 1.73 nm, which matches the distance required for covalent porphyrin dimerization. This forces proximity bring the bromine terminal group into close contact, forming a topochemical configuration that favors subsequent Ullmann coupling [77]. In another example, brominated anthraquinone derivatives utilize carboxyl hydrogen bonds to form dimers, which further stack into columnar structures through π-π interactions. The resulting interlayer spacing of 0.35 ± 0.02 nm positions adjacent bromine atoms in close proximity, creating a structural environment conducive to light-induced dehalogenation and radical coupling [78]. Additionally, 9-diazoanthracene (DAF) molecules on Ag(111) assemble into chiral dimers through hydrogen bonding between the diazo group and a neighboring aromatic hydrogen atom, providing a well-defined initial configuration for subsequent photolysis or non-elastic electron tunneling to generate carbenes intermediates (Figure 9f) [21].

### 4.2. Interface Engineering and Substrate Tailoring

As detailed in Section 2.3, the electronic density of states of the substrate fundamentally dictates the photoexcitation lifetime and accessible pathways. While metallic surfaces facilitate efficient substrate-mediated processes, the inherent electronic coupling often leads to rapid quenching of excited states [44]. To circumvent this, insulating platforms [34] or thin decoupling layers are strategically employed to preserve excited-state lifetimes, enabling “gas-phase-like” direct photochemistry.

Beyond the simple choice of substrate, surface design, drawing on decades of expertise in tailoring on-surface reactions, has emerged as a powerful tool for light-driven synthesis. By constructing atomic-scale interfacial layers such as surface oxides, nitrides, and sulfides, researchers can precisely manipulate carrier dynamics and energy alignment. A typical example is the use of thin oxide films, such as Al_2_O_3_ on Ni_3_Al(111) [79], which provides a well-defined template expected to stabilize photo-generated intermediates that would otherwise be short-lived on bare metals surfaces [80]. This transition from passive supports to actively engineered interfaces enables the stabilization of fragile molecular backbones and the realization of high chemoselectivity, representing a frontier in the architectural precision of on-surface photochemistry.

### 4.3. Wavelength and Polarization Tuning

The energy and polarization of incident light serve as external handles for selectively accessing different reaction pathways in surface photochemistry [81]. By tuning these parameters, it is possible to target specific electronic transition or interfacial processes, thereby achieving precise control over reaction mechanisms and outcomes.

Wavelength-dependent studies provide a systematic means to distinguish among competing excitation channels. A well-documented example is the photodebromination of DBTP on Au(111), where the reaction efficiency follows the order 313 nm > 405 nm > 700–750 nm. This trend reflects a progression from direct molecular excitation (313 nm, matching the molecular HOMO–LUMO gap) to hot electron-mediated processes (405 nm) and interfacial charge transfer (700–750 nm) [7,33]. Similar wavelength selectivity has been observed in other systems. For instance, the photodissociation of dimethyl disulfide ((CH_3_S)_2_) on Cu(111) and Ag(111) exhibits a distinct reaction peak at 450 nm, where the substrate absorption is negligible, ruling out hot electron mechanisms and pointing instead to direct excitation enabled by molecule–substrate hybridization [82]. In addition to wavelength selection, the polarization state of light provides an additional dimension for distinguishing different photochemical excitation mechanisms [83]. On Au(111), angle-dependent measurements the DBTP photoreaction yield under p- and s-polarized light (0–80°) reveal that the efficiency under p-polarization follows the calculated absorbance profile of the gold surface, with a maximum at approximately 65.9°. This behavior deviates from the dipole-angle dependence predicted for direct molecular excitation (maximum around 50°), providing clear evidence for a substrate-mediated excitation pathway. Polarization-dependent two-photon photoelectron spectroscopy (2PPE) on the phenol/Ag(111) system further corroborates such substrate-mediated mechanism [76].

By simultaneously controlling the wavelength and polarization direction of the incident light, the selectivity of surface photoreactions can be further enhanced, allowing for precise control over the reaction pathways. In the Fantrip/passivated graphite system, the transition dipole moment of the anthracene unit is oriented parallel to the molecular short axis. By using p-polarized light with a 45° incidence angle, the light field is aligned with this dipole, maximizing absorption efficiency while minimizing thermal side effects [36,68]. In azide-based systems on Au(111), wavelength selectivity is equally pronounced. Exposure to 360 nm and 266 nm light led to over 90% conversion within minutes, while 450 nm light exposure yields only about 26% conversion after one hour, highlighting the importance of matching photon energy to the target electronic transition [84].

**Figure 10:**
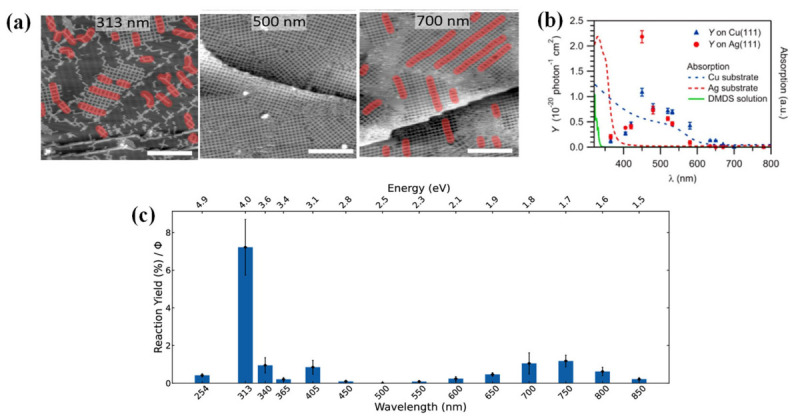
Schematic of the mechanism of wavelength- and polarization-dependent photochemical reactions on metal surfaces. (**a**) Typical scanning tunneling microscope (STM) images of DBTP after exposure to light at different wavelengths, with products highlighted by red overlays [33]; (**b**) Wavelength (λ) dependence of photodissociation yield (Y), where Y equals the rate constant (k) divided by the number of incident photons per second [80]; (**c**) Wavelength-dependent reaction efficiency of DBTP on Au(111), showing the highest activity at 313 nm, with residual activity remaining in the near-infrared region [33].

Overall, wavelength and polarization offer complementary control parameters for on-surface photochemistry. By matching photon energy with the molecular or interfacial electronic states and adjusting the light field with transition dipole moments, different excitation pathways can be selectively activated or suppressed. These include direct molecular excitation, hot electron transfer, and interfacial charge transfer. This multi-parameter approach not only deepens mechanistic understanding but also offers a practical strategy for developing highly selective on-surface synthetic methods (Figure 10).

## 5. Comparison: Thermal vs. Photo-Induced Synthesis

### 5.1. Bond Activation and Chemoselectivity

Thermal activation relies on increasing the kinetic energy of molecules to overcome bond dissociation barriers. However, the elevated temperatures required for cleaving target bonds, including C-X (X = Br, Cl, I) or C-H, often exceed the thresholds for undesired processes [85,86,87]. Consequently, side reactions including cyclodehydrogenation, molecular desorption, and surface defect formation are frequently observed [88,89,90]. For example, thermal dechlorination of aryl chlorides on the Au(111) surface requires temperatures up to approximately 670 K. These ultrahigh thermal conditions not only drive the cleavage of C–Cl bonds but also readily induce cyclodehydrogenation that precedes dehalogenation and intermolecular polymerization. As a result, a complex mixture of uncontrollable byproducts is formed, including cyclodehydrogenated aromatic derivatives, randomly polymerized oligomers and polymers, fragmented small molecules, and carbon–metal hybrid defective byproducts from molecular desorption and surface lattice damage [14].

**Figure 11:**
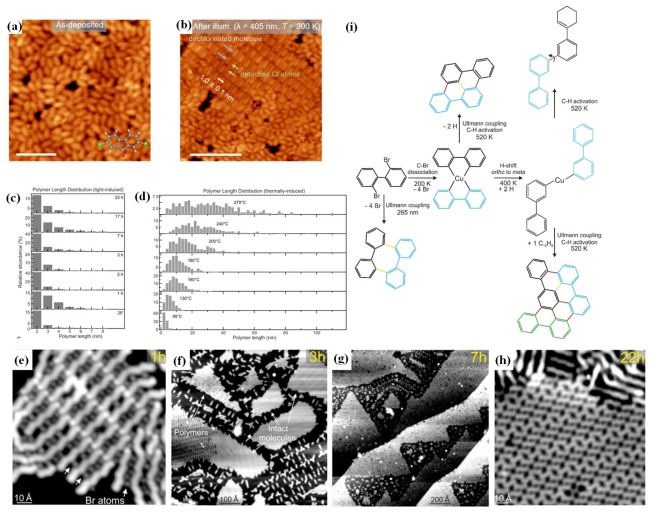
(**a**) STM image after depositing DCF molecules on Au(111) at room temperature (Vs = −1.5 V, It = 100 pA, scale bar: 4 nm). The chemical model of DCF is shown at the bottom right. (**b**) STM image after illuminating the DCF-covered sample with a violet light at 405 nm for 15 h at room temperature (Vs = −1.8 V, It = 100 pA, scale bar: 7 nm). Gray arrows depict dechlorinated DCF molecules, while the green arrows indicate the detached chlorine. White arrow represents the distance between adjacent molecules [14]. (**c**) Polymer length distribution after UV (266 nm) laser light illumination; (**d**) Polymer length distribution as a function of the sample annealing temperature; (**e**–**h**) Illumination with UV light. Scanning tunneling microscopy images of 0.5 ML DBA/Au(111) taken at 7.5 K: UV (266 nm) laser light illumination at room temperature (3 h (**f**) and 7 h (**g**)); 2D close-packed oligomers after 1 h of illumination (**e**); Zoom-in of a 2D close-packed arrangement of intact DBA molecules after 22 h of illumination (**h**) [10]; (**i**) Reaction Scheme for 2,2′-Dibromobiphenyl on Cu(111): Black, Blue, and Green Mark Different Reactant Molecules [12]. Bonds formed by Ullmann coupling and C–H activation are marked in yellow and red, respectively.

Photo-induced activation offers a fundamentally different approach [91]. By delivering energy selectively through resonant excitation of specific bonds or electronic states, it enables bond cleavage at cryogenic or room temperature while leaving other functional groups intact. A striking illustration is the photo-induced dechlorination of 2,7-dichloro-9-fluorenone (DCF) on Au(111) [14]. Upon 405 nm irradiation at room temperature, the C-Cl bonds are cleaved with high selectivity, while the C=O bond remains unaffected. The resulting dechlorinated monomers self-assemble into ordered brick-type nanostructures, a level of order not achieved under thermal conditions (Figure 11a,b) [14].

### 5.2. Polymer Length and Defect Density

Thermal and photo-induced syntheses produce polymer architectures with distinct morphological characteristics [90]. In thermal activated processes, polymer length is governed by molecular diffusion, which increases with temperature [92]. For 2,6-dibromoanthracene (DBA) molecules on Au(111) surfaces, raising the annealing temperature from 95 °C to 270 °C leads to a linear increase in polymer length from approximately 5 nm to over 30 nm (Figure 11d). The resulting structures consist of linear chains with occasional kinks, which can aggregate into closely packed island-like structures [10].

Photo-induced synthesis, by contrast, operates under conditions where diffusion is severely limited. For the same DBA system, 266 nm UV illumination at room temperature yields oligomers composed exclusively of 2 to 4 anthracene units. The polymer length distribution remains narrow and stable even when illumination is extended 1 h to 22 h, with no detectable formation of longer chains (Figure 11c,e–h). This behavior reflects a reaction regime where bond activation occurs without significant molecular mobility, yielding uniform short-chain products with low defect density [10].

### 5.3. Reactive Intermediates Under Mild Photo-Conditions

The ability to generate and stabilize reactive intermediates chemical species is pivotal for understanding reaction mechanisms and for developing novel synthetic strategies [19]. Thermal activation often drives intermediates toward further reactions or decomposition, making them difficult to observe or isolate. On Cu(111), for instance, thermal annealing of 2,2′-dibromobiphenyl leads to the formation of stable C–Cu bonded organometallic intermediates that subsequently undergo uncontrolled transformations (Figure 11i).

Photoexcitation under cryogenic conditions offers a powerful alternative. Using 265 nm ultraviolet light, which matches the π → π* transition of the same precursor, C–Br bonds are cleaved selectively without forming C–Cu bonds or activating other functional groups. The resulting aryl radical intermediates are stabilized by the low-temperature environment, which suppresses diffusion and prevents side reactions. Throughout the photolytic process, only transient radical are present, and the final product, tetraphenylene, is obtained as a single species with no detectable byproducts (Figure 3b) [12].

## 6. Conclusions and Future Outlook

In summary, light-driven on-surface synthesis has emerged as a powerful complement to traditional thermal activation, enabling precise, chemoselective, and low-temperature fabrication of low-dimensional nanostructures. By leveraging distinct photoexcitation pathways, including direct molecular excitation, substrate-mediated hot electron transfer, and interfacial charge transfer, these photochemical approaches allow unprecedented control over bond activation, polymerization pathways, and molecular isomerization while minimizing side reactions and defects. Strategic manipulation of molecular preorganization, surface passivation, and light parameters including wavelength and polarization further enhances reaction selectivity and efficiency, providing a versatile platform for constructing one- and two-dimensional covalent architectures.

Looking forward, the integration of advanced light field modulation, rational substrate engineering, and bespoke molecular design holds great promise for addressing current challenges in scalability and complexity. Continued efforts in these directions are expected to enable the controlled synthesis of functional nanomaterials with tailored properties, as well as the development of dynamic, light-responsive molecular devices. Such advancements will mark significant progress toward the realization of programmable surface chemistry and next-generation nanofabrication technologies.

## Data Availability

No new data were created or analyzed in this study.
